# Gender Differences in Neuropsychological Performance across Psychotic Disorders – a Multi-Centre Population Based Case-Control Study

**DOI:** 10.1371/journal.pone.0077318

**Published:** 2013-10-28

**Authors:** Jolanta Zanelli, Kevin Morgan, Paola Dazzan, Craig Morgan, Manuela Russo, Izabela Pilecka, Paul Fearon, Arsime Demjaha, Gill A. Doody, Peter B. Jones, Robin M. Murray, Abraham Reichenberg

**Affiliations:** 1 Department of Psychosis Studies, Institute of Psychiatry, King’s College London, London, United Kingdom; 2 Department of Psychology, Westminster University, London, United Kingdom; 3 Centre for Public Mental Health, Health Service and Population Research Department, Institute of Psychiatry, King’s College London, London, United Kingdom; 4 Department of Psychiatry, University of Cambridge, Cambridge, United Kingdom; 5 Division of Psychiatry, University of Nottingham, Nottingham, United Kingdom; The University of Queensland, Australia

## Abstract

**Background:**

Patients with schizophrenia and other psychoses exhibit a wide range of neuropsychological deficits. An unresolved question concerns whether there are gender differences in cognitive performance.

**Methods:**

Data were derived from a multi-centre population based case-control study of patients with first-episode psychosis. A neuropsychological test battery was administered to patients with a diagnosis of schizophrenia or schizoaffective disorder (N=70, 36% females), bipolar/mania (N=34, 60% females), depressive psychosis (N=36, 58% females) and healthy controls (N=148, 55% females). Generalized and specific cognitive deficits were compared.

**Results:**

There was strong evidence for disorder-specific gender differences in neuropsychological performance. Males and females with schizophrenia showed similar pervasive neuropsychological impairments. In psychotic depressive disorder females performed worse than males across neuropsychological measures. Differences in neuropsychological performance between males and females with bipolar/manic disorder were restricted to language functions. Symptom severity did not contribute to the observed gender differences.

**Conclusions:**

Early in the course of psychotic illness, gender related factors appear to moderate the severity of cognitive deficits in depressive psychosis and bipolar/mania patients.

## Introduction

The existing literature suggests that there are pronounced sex differences in schizophrenia, including differences in age of onset, clinical symptoms, brain structure and outcome [1,2]. For example, on average, males have earlier age of onset, and longer and more frequent acute psychotic episodes, while females show better premorbid functioning, more positive symptoms, less negative symptoms, and better response to antipsychotic medication [2,3]. Therefore studying gender effects may help explain differences in clinical presentation, disease course, and response to both pharmacological and psychosocial treatment. 

It is well established that patients with schizophrenia and other psychosis exhibit a wide range of neuropsychological deficits [4,5]. An unresolved question, however, is whether there are gender differences in cognitive performance. In terms of neuropsychological function, evidence concerning schizophrenia is mixed. While some studies report less severe cognitive impairments in female patients on most cognitive domains [6,7], other studies found that females exhibited greater cognitive deficits [8,9]. Some research failed to find any significant gender differences in cognition in schizophrenia [4,10-13], and yet others reported domain-specific gender differences, with males showing greater impairments than females in sustained attention, language, executive function and intelligence [6,14-16]. 

The question of gender differences in neuropsychological function in other psychotic disorders has rarely been examined. Carrus et al [17] reported that in bipolar patients, males underperformed females on immediate memory tasks (encoding and retrieval processes). Barret et al [18] also noted that male patients underperformed female patients in measures of spatial working memory. These studies did not find gender differences in performance on measures of planning, attentional set-shifting, verbal fluency, general intellectual ability, response inhibition and other executive functions. 

However most of the studies that examines neuropsychological function in psychosis by gender examined patients with established illness and thus performance may have been impaired by issues concerning treatment or illness course. The present study examined gender differences in neuropsychological function in first episode patients with a range of psychotic illnesses, including schizophrenia, bipolar/manic and depressive psychoses. Four features of the present study are especially important. First, this study examines a large epidemiological cohort of first-episode psychosis patients with neuropsychological data. Second, a large community control sample was epidemiologically ascertained, giving detailed and reliable norms for comparison. Third, a comparison across diagnostic groups is a unique feature of the present study. Finally, both patient and comparison samples are ethnically diverse, thus increasing the potential generalisability of our results.

The main objective is to investigate whether there are gender differences in the profile of neuropsychological performance in patients with first episode of psychosis. Because of the conflicting results in previous research, which examined cognition particularly in schizophrenia, no a priori hypothesis regarding gender differences in neuropsychological performance was made. The study describes the methodology and presents the findings of the investigation with the aim of developing a greater understanding of the gender differences particularly in affective disorders.

## Methods

### Study Population

The data were derived from the baseline AESOP (Aetiology and Ethnicity in Schizophrenia and Other Psychoses) study, a population based case-control study of first-episode psychosis. Ethical approval for the AESOP study was obtained from the Nottingham, Bristol and London Local Research Ethics Committees. Informed consent was sought from all participants, using an information sheet and consent form approved by the above noted research ethics committees. Refusal to participate did not in any way affect the care of those approached. All participants had capacity to consent.

The AESOP study is a multi-centre population based incidence and case-control study conducted in the UK. Its primary aim is to investigate the high rates of psychosis in African-Caribbean populations from the UK, and from this to shed light on the aetiology of psychosis in general.

 In the case of psychiatric patients, permission to invite them to participate was first sought from the treating clinician. In the case of healthy controls all were judged to have capacity to make a decision to participate by the researcher seeking consent. Capacity to consent was established by either having a non-member of the research team obtain consent and/or making sure that the participants understands the study by him/her explaining the study to the person obtaining consent. The study identified all cases aged 16-64 years with a first episode of psychosis (F10-F29 and F30-F33 in ICD-10) who presented to specialist mental health services in tightly defined catchment areas in South-East London, Nottingham (between September 1997 and August 2000) and in Bristol over a nine-month period (September 1997 to May 1998). The South London catchment area comprised the former Bethlem and Maudsley NHS Trust areas served by the Maudsley Hospital and the former South Western Hospital. Those catchment areas represent a large area of South London encompassing the boroughs of Lambeth, Southwark and Croydon. Nottingham has well-circumscribed area served by a single provider of mental health services (Nottingham Healthcare NHS Trust). Finally, the Bristol area comprised the well-defined catchment area of the Avon and Wiltshire Mental Health Partnership NHS trust. Exclusion criteria were previous contact with health services for psychosis, organic causes of psychotic symptoms, transient psychotic symptoms due to acute intoxication (as defined by ICD-10), and an IQ of less than 50. We excluded participants with IQ <70 to ensure reliability of neuropsychological testing The study further included a random sample of controls with no past or present psychotic disorder, who were recruited using a sampling method that matched cases and controls by area of residence. A detailed overview of the AESOP design and methods has been published elsewhere [19]. 

### The Analytic Cohort

**Figure 1:**
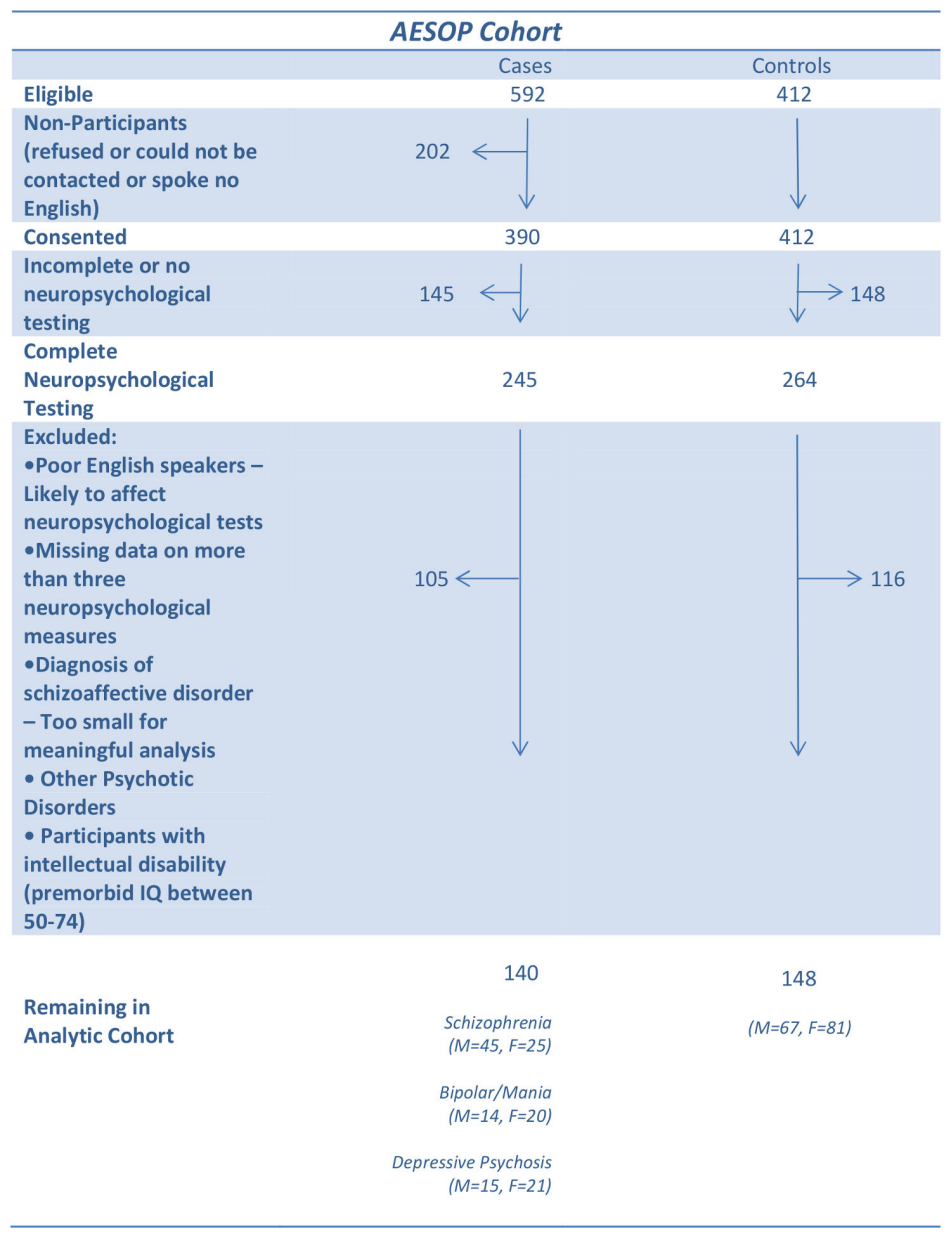
The Analytical Cohort.

The derivation of the sample included in the present analysis is illustrated in Figure **1**. The analytic cohort comprised of male and female patients who had a consensus ICD-10 diagnosis of schizophrenia or schizoaffective disorder (M=45, F=25), bipolar/mania (M=14, F=20), depressive psychosis (M=15, F=21) and 148 healthy controls (M=67, F=81). For all cases and controls there were complete interview data as well as: a) IQ measurements on the National Adult Reading Test (NART) [20], and a short form of the Wechsler Adult Intelligence Scale – Revised (WAIS-R) [21]; and b) a neuropsychological battery (see below). Cases and controls were required to be native speakers of English or to have migrated to the UK by age 11. The latter requirement ensured that each participant had a good command of English as a non-native language as they would have completed at least their secondary education in the UK, thus minimising the effect of linguistic or cultural biases on the performance of a multi-ethnic sample. In accordance with previous studies [2], cases or controls with missing data on more than three neuropsychological measures were excluded. 

### Diagnostic Assessment and Symptom Data

Clinical data were collected using the Schedules for Clinical Assessment in Neuropsychiatry (SCAN) [22,23]. The SCAN incorporates the Present State Examination Version 10, which is used to elicit symptom-related data at the time of presentation. Our ratings on the SCAN were based on clinical interview supplemented by case note review and information from informants (e.g. health professionals, close relatives). Researchers were trained in the SCAN interview on a World-Health-Organization (WHO) approved course and established pre-study reliability using independent rating of videotaped interviews. ICD-10 diagnoses were determined using the SCAN data on the basis of consensus meetings involving one of the principal investigators and other members of the research team. 

Symptom ratings were calculated according to the SCAN’s Item Group Checklist (IGC) [22]. The IGC combines scores from several SCAN items specific to a particular group of symptoms. For example: the IGC item, *Special features of depressed mood* includes feeling of loss, loss of reactivity, morning depression, preoccupation with death or catastrophe, pathological guilt, guilty ideas of reference, loss of self-esteem and dulled perception. Scores for individual item groups ranged from 0 (absent) to a maximum of 2 depending on the frequency and severity of symptoms. Following a recent factor analysis on data from this sample [24], individual item groups were aggregated into five symptoms dimensions: ***NegativeSymptoms*** (Flat and incongruous affect, Poverty of speech, Motor retardation and Catatonic behaviour, and Nonverbal communication); ***RealityDistortion*** (Bizarre delusions and interpretations, Delusions of reference, Delusions of persecution, Experience of disordered form of thoughts, Nonspecific auditory hallucinations, and Non-affective auditory hallucinations), ***DepressiveSymptoms*** (Depressed mood, Depressive delusions and hallucinations, and Special features of depressed mood); ***Disorganization*** (Incoherent speech and Emotional turmoil) and ***ManicSymptoms*** (Heightened subjective functioning, Expansive mood, Rapid subjective tempo, Expansive delusions and hallucinations, Overactivity and Socially embarrassing behaviour).

### Neuropsychological Assessment

Sixteen neuropsychological measures were selected to assess six broad cognitive domains: (1) Learning and Memory (verbal and visual); (2) Executive functions and working memory; (3) Attention, concentration and mental speed; (4) Language; (5) Visual constructual/perceptual abilities, and (6) WAIS-R verbal intelligence. ***LearningandMemory*** were assessed using trials 1 through 5 (learning), trial 6 (immediate recall), and trial 7 (delayed recall), respectively, of the Rey Auditory Verbal Learning Test (RAVLT), and the Visual Reproduction sub test of the Wechsler Memory Scale - Revised (WMS-R) [25]. ***Executivefunctionsandworkingmemory*** were evaluated using the Trail Making Test - part B, Letter-Number Span, and Sets AB and B of Raven’s Coloured Progressive Matrices (CPM). ***Attention,concentrationandprocessingspeed*** were measured using Trail Making - part A, and the WAIS-R Digit Symbol subtest. ***Language*** was evaluated using the Category (Semantic) Fluency (categories ‘body parts’, ‘fruits’ and ‘animals’), and Letter Fluency (letters F, A, S). ***Visual-Spatialperceptionandorganisation*** were assessed using set A of Raven’s Coloured Progressive Matrices and the WAIS-R Block Design. ***WAIS-Rverbalintelligence*** was assessed by the WAIS-R Vocabulary and Comprehension sub tests. For a detailed description of measures see Spreen & Strauss, 1991 [26]. Premorbid intelligence was estimated using the National Adult Reading Test (NART) [20], Full-scale IQ was derived from the WAIS-R [27] subtests in the neuropsychological testing battery [1]. All tests were administered and scored by specially trained research workers. Patients were tested as soon as possible following presentation and entry into the study. At the time of neuropsychological assessment all patients were adjudged to be clinical stable for the purposes of testing.

### Creating gender-specific norms for neuropsychological tests

A regression based approach was used to create normative standards for the neuropsychological tests. This was done separately for males and females. Age, ethnicity and education were regressed on each of the neuropsychological measures in the healthy sample of males and females. Then, scores were adjusted on the basis of these regression results and standard (i.e., Z) scores were created. The regression corrected scores were also inspected for both skewness and kurtosis. Only the Raven’s test and Trail Making Test part A & B had significant skewness and these variables were log-transformed before standardisation. The same adjustment and standardisation procedure was applied to the male and female patient sample, using the normative standards from the control sample.

### Data analysis

SPSS (Statistical Package for the Social Sciences) version 15 software was used to analysed data. Gender differences in socio-demographic characteristics, symptom severity and measures of IQ among the three diagnostic groups were assessed using Analysis of Variance (ANOVA) models, Pearson Chi-Square or Fisher’s Exact Test when appropriate. 

Individual neuropsychological measures were grouped into the six broad domains described in the section on Neuropsychological Assessment, and these domains were used in the ANOVA models described below. Domain scores were computed, separately, for males and females, as the average of the normative adjusted scores. For descriptive purposes we carried the following *a-priori defined* comparisons: for each diagnostic group male and female patient domain scores were compared to the performance of the males and females in the control group using ANOVA models. Gender differences in neuropsychological performance in the three diagnostic groups were examined using a Repeated Measures Analysis of Variance (ANOVA) model. Due to missing data on individual tests, degrees of freedom varied slightly. ANOVA was used to examine gender differences in the following symptom dimensions: negative symptoms, reality distortion, depressive symptoms, disorganization and manic symptoms [4].

## Results

### Demographic Characteristics

Table **1** presents the demographic characteristics of male and female patients in the three diagnostic groups and healthy controls. The groups differed significantly in gender distribution (χ^2^=9.98, df=3, p=0.03), with a higher proportion of males in the schizophrenia group compared with the bipolar/manic and depressive psychosis groups. Gender differences in ethnic origin were only evident in the bipolar/manic group (χ^2^=5.55, df=2, p<0.018), with higher proportion of non-white females. Age of onset was higher for females compared with males in the schizophrenia group (t=-2.58, p=0.012). There were no significant gender differences in level of education.

**Table 1:** Demographic Characteristics of Males and Females Patients with First-Episode of Psychosis and Healthy Controls.

	**Schizophrenia**		**Bipolar/Manic**		**Depressive Psychosis**		**Controls**	
	*Males*	*Females*	*Males*	*Females*	*Males*	*Females*	*Males*	*Females*
	(N=45)	(N=25)	(N=14)	(N=20)	(N=15)	(N=21)	(N=67)	(N=81)
**Age (*±SD*)**	24.9±6.5	30.4±11.4	26.0±5.8	30.9±8.7	33.2±12.6	40.5±12.7	39.22±13.27	38.89±12.45
**Years of Education**								
*11 (compulsory*)	64.4%	60.0%	35.7%	50.0%	53.3%	76.2%	38.8%	55.6%
*12-13 (post-compulsory*)	28.9%	20.0%	50.0%	15.0%	20.0%	14.3%	32.8%	27.2%
*14 (college, graduate, post-graduate*)	6.7%	20.0%	14.3%	35.0%	26.7%	9.5%	28.4%	17.2%
**Ethnicity** *Caucasian*	60.0%	60.0%	85.7%	45.0%	60.0%	66.7%	70.1%	81.5%

### Current and Estimated Premorbid IQ

**Figure 2:**
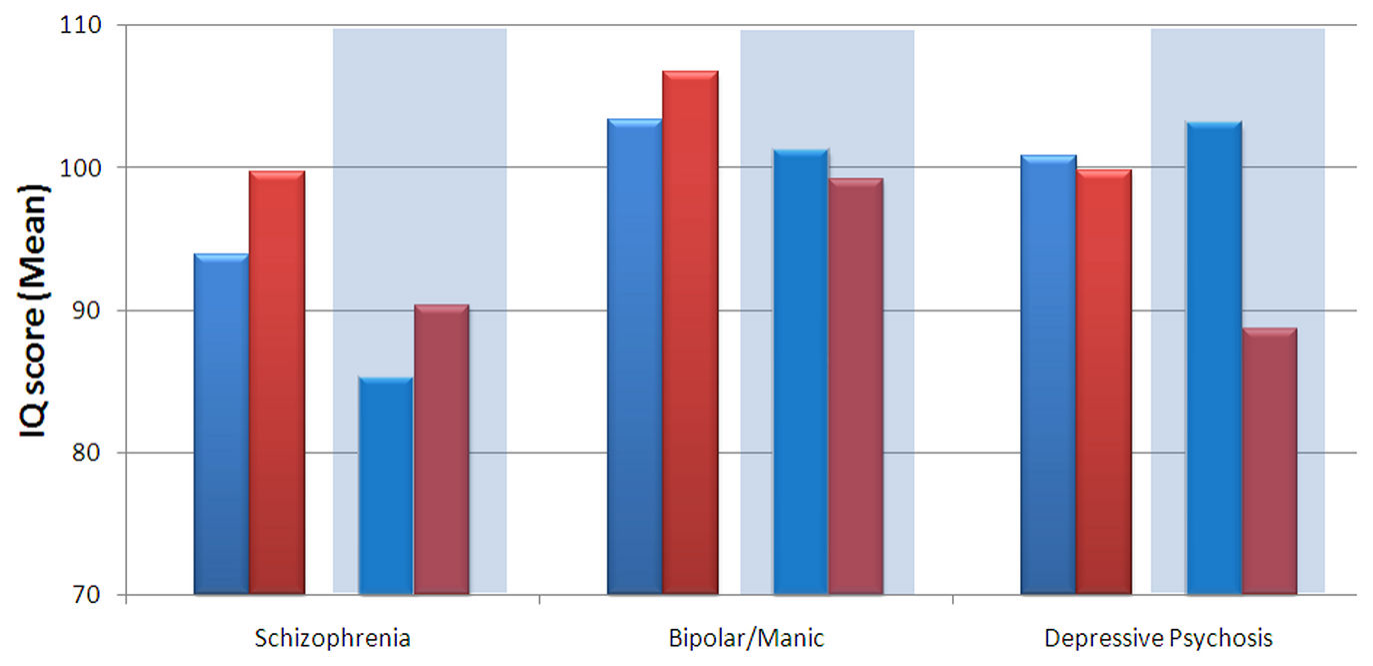
Estimated Premorbid (Non-shaded Areas) and Current (Shaded Areas) IQ in Males (Blue Bars) and Females (Red Bars) across Diagnostic Groups: Schizophrenia (N=45 Males, N=25 Females); Bipolar/Manic (N=14 Males, N=20 Females), and Depressive Psychosis (N=15 Males, N=21 Females).

Male and female current and estimated premorbid IQ in the three diagnostic groups are shown in Figure **2**. Males and females were compared to controls on current and estimated premorbid IQ measures using ANOVA models. The significance level was adjusted for multiple testing by Bonferroni correction and was conservatively set at p≤0.0125 (0.05/4). 

### Comparison to controls

In the schizophrenia group, females performed significantly worse than healthy controls only on current IQ, whereas males performed significantly worse on both current and estimated premorbid IQ. In the bipolar/manic group there were no significant gender differences. In the depressive psychosis group, females performed significantly worse on current IQ compared with controls.

### Comparison of males and females within diagnostic groups

Analysis of variance (ANOVA) revealed a significant effect of diagnosis on current IQ (F=7.298, df=2, 134, p=0.001), but no significant effect of gender (F=1.674, df=1, 134, p=0.198). However, there was a significant gender by diagnosis interaction effect (F=4.216, df=5, 134, p=0.017), where females in the depressive psychosis group exhibited lower scores than males. There was a significant effect of diagnosis on premorbid IQ (F=3.912, df=2, 129, p=0.022) but no significant effect of gender (F=1.171, df=1, 129 p=0.281), or gender by diagnosis interaction (F=0.674, df=5,134, p=0.511). 

### Neuropsychological Performance

**Figure 3:**
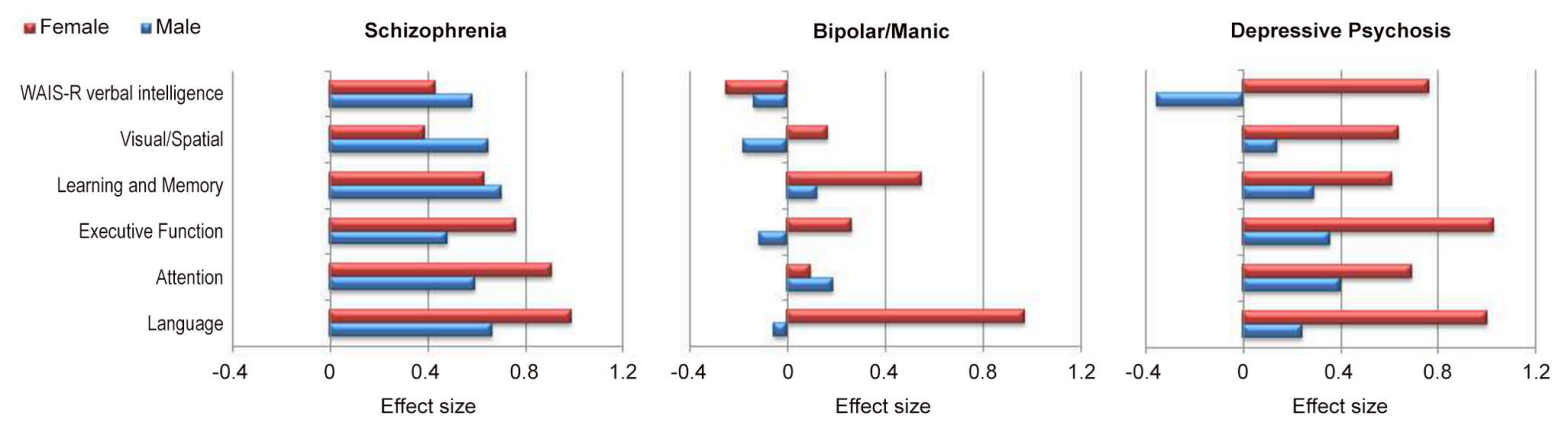
Effect Size Comparing Neuropsychological Functioning in Males (Blue Bars) and Females (Red Bars) according to Diagnostic Groups: Schizophrenia (N=70; 45 Males, 25 Females); Bipolar/Manic (N=14 Males, N=20 Females), and Depressive Psychosis (N=15 Males, N=21 Females) *Note: Impairments are represented by positive values on scale*.

Figure **3** presents effect size comparing neuropsychological functioning in males and females for each of the three diagnostic groups. 

### Comparison to controls

Males and females were compared to controls on each neuropsychological domain using ANOVA models. The significance level was adjusted for multiple testing by Bonferroni correction and was conservatively set at p≤0.008 (0.05/6). In the schizophrenia group, females performed significantly worse than healthy controls on learning, attention, executive function and language domains whereas males performed significantly worse on all domains. In the bipolar/manic group, females performed significantly worse than healthy controls on neuropsychological domains assessing language and learning, whereas males performance was similar to that of controls. In the depressive psychosis group females performed significantly worse than controls on all neuropsychological domains. There were no significant differences between males and healthy controls.

### Comparison of males and females within diagnostic groups

A repeated-measures ANOVA of gender, diagnosis and neuropsychological domain showed a significant effect of gender (F=5.08, df=1, 112, p=0.026) and of diagnostic group (F=4.342, df=2, 112, p=0.001), but no significant gender by diagnosis interaction (F=1.458, df=2, 112, p=0.237). However, there was a significant three-way interaction between gender, diagnosis and neuropsychological domain (F=2.123, df=10, 112, p=0.021), indicating that the profile of neuropsychological impairment varies as a function of both diagnosis and sex. To further examine the three way interaction in detail, we repeated the analysis separately for each diagnostic group. In the schizophrenia group there was no significant effect of gender (F=0.079, df=1, 55, p=0.78) and no significant gender by neuropsychological domain interaction (F=1.904, df=5, 55, p=0.094) (Figure **3**). Differences between males and females in neuropsychological performance were small - not greater than 0.3 standard deviation (ES<0.3). Females performed worse than males on language, attention and executive function domains and better than males on WAIS-R verbal intelligence and visual-spatial domains. In the bipolar/manic group there was no significant gender effect (F=1,604, df=1, 25, p=0.217) but there was a significant gender by neuropsychological domain interaction (F=2.854, df=5, p=0.018). Females performed worse than males on language, executive function, learning and memory, and visual/spatial domains with effect size of difference ranging between 0.2 to 1. Post hoc contrasts revealed a significant difference on the language domain (t=2,37, df=25, p=0.026), with females performing worse than males (ES=-1.05). In the depressive psychosis group there was a significant effect of gender (F=5.729, df=1, p=0.023) but no significant gender by neuropsychological domain interaction (F=1.229, df=5, 32, p=0.298). Females performed worse than males on all neuropsychological domains (Figure **3**) with effect sizes of difference ranging from 0.6 to 0.4. 

### Symptom severity and neuropsychological performance

We examined gender differences in symptom severity in these diagnostic groups (Table **2**). There were no significant gender differences in symptom severity in any diagnostic group, with the exception of disorganisation symptoms in schizophrenia where males had more severe symptoms than females (p=0.038). We also examined the associations between symptoms severity and neuropsychological performance in males and females in each diagnostic group. No consistent pattern of association emerged. The overwhelming majority of correlation coefficients were small and not statistically significant (data available on request).

**Table 2:** Descriptive Statistics for Test of Gender Differences between Neuropsychological Scores and Symptom Dimensions.

	**Schizophrenia**		**Bipolar/Manic**		**Depressive Psychosis**	
	*Males*	*Females*	*Males*	*Females*	*Males*	*Females*
	(N=45)	(N=25)	(N=14)	(N=20)	(N=15)	(N=21)
**Symptom Dimensions**						
*Manic Symptoms*	1.6±2.6	1.7±2.8	6.2±3.3	5.6±3.3	3.3±4.1	0.3±0.5
*Depressive Symptoms*	1.4±1.7	1.6 ±2.0	1.1±1.6	0.8±1.6	3.1±1.6	3.5±1.5
*Reality Distortion*	4.1±2.3	3.8±2.1	2.2±2.6	3.8±1.0	3.0±2.0	3.6±1.7
*Negative Symptoms*	2.2±2.2	1.7±2.8	0.4±0.5	1.1±1.8	2.1±1.7	1.9±2.0
*Disorganization*	1.3±0.9	0.6±0.7	0.7±1.5	0.3±0.5	0.1±0.3	0.5±0.9

## Discussion

To the best of our knowledge, this is the first population-based epidemiological study of gender differences in neuropsychological performance across a range of psychotic disorders. The study provides new evidence to what is known about the cognitive impairment in psychoses in two important ways. First, while both males and females with schizophrenia showed pervasive neuropsychological impairments, only females with bipolar/manic or psychotic depressive disorders showed significant impairments compared with healthy controls. Second, the study shows that gender differences in neuropsychological functions are disorder specific. Gender differences were evident in psychotic depression but not in schizophrenia. There was also evidence of a gender based domain- specific impairment in bipolar/manic disorder. Females performed significantly worse than males on the language domain. Gender differences were not due to differences in symptom severity. 

Both males and females with schizophrenia showed generalized and clinically severe cognitive impairments. We found no statistically significant gender differences in neuropsychological performance in schizophrenia. Previous studies reported mixed results [6,12,16], Hoff et al [12] studying first episode and chronic schizophrenia patients reported that after controlling for symptom severity there were no sex differences in cognition between male and female patients with schizophrenia. Other studies reported domain-specific gender differences with males showing greater impairments than females in sustained attention, language, executive function and intelligence [6,14,16]. The results of the present study indicate that the well replicated finding of a severe cognitive impairment in schizophrenia is equivalent in males and females [28-30]. 

In the bipolar/manic group, males showed no cognitive impairments. Gender differences were evident in the language domain, where females performed statistically significantly worse than males. The impairment in the language domain was very large (more than 1SD compared to controls). Whereas, Barret et al [18] and Carrus et al [17] found that males underperformed females on immediate memory and spatial working memory tasks, we saw no evidence for gender differences on related neuropsychological domains. The studies by Barret et al [18] and Carrus et al [17] however, used clinical, not epidemiological samples and studied older and more severe patient samples. 

We are not familiar with other studies examining gender differences in neuropsychological functioning in patients suffering from depressive psychosis. Our data showed distinct cognitive patterns in this patient population. Females were impaired across all neuropsychological domains compared to males. Males showed relatively preserved cognitive ability. 

One possible explanation for our finding could be female-specific vulnerability to the effects of psychotic illness and medication. Antipsychotic medications have been shown to adversely affect cognition [31]. However, severity of neuropsychological impairments in females was not identical across domain and disorders. Therefore a complex interaction of medication and vulnerability would be required in order to support this explanation. Alternatively, future diagnostic shift may help explain some of the findings. Patients were assessed 6 months after their first admission. Previous research had shown that while a schizophrenia diagnosis is very stable, 20% of bipolar and 30% of major depression will shift diagnosis to schizophrenia spectrum within 24 months of their first admission [32,33]. One could speculate that if such a diagnostic shift was more likely to be found in female patients it could help explain the findings. 

This study has several strengths. It is a large, first-episode psychosis study incorporating a population-based epidemiological sample. A limitation of most previous studies is that they used clinical rather than epidemiological samples. Clinical samples involve patient series, not complete populations. As a result, it is difficult to generalize from studies using clinical samples. The fact that we included a large healthy comparison group also strengthens the interpretation of our results. However, the following limitation of the study should also be acknowledged. Although the overall sample size is large groups by gender analyses had small group size. Nevertheless our interpretation of results relies not only on statistical significance but also on effect size and both are reported. Although there are statistically significant gender differences in cognition in the normal population, these gender effect sizes are small, and because within gender variability is greater than between gender variability in normal subjects, having within gender comparison groups increases the statistical power to detect gender differences in patients. Also, we had insufficient information on medication to reliably determine dosage, and no patients were drug naive. Medication type and side effects can influence subjects' neuropsychological performance. Nonetheless, we observed only weak correlations between neuropsychological functioning and symptom severity, and these could not explain the observed results. 

Furthermore, the information about drug consumption was not collected therefore we cannot determine any relationship between the drug use and its impact on psychosis. 

In summary, the results of the present study indicate that early in the course of psychotic illness, gender related factors appear to moderate the severity of cognitive deficits in bipolar/mania and depressive psychosis patients. In contrast, gender related factors have only minimal effects on the neuropsychological impairment in schizophrenia. Such knowledge may be valuable in designing cognitive-targeted intervention in psychosis. 
